# Secret Remedies and Branded Products—VI

**Published:** 1908-05-30

**Authors:** 


### Therapeutics.

### SECRET REMEDIES AND BRANDED PRODUCTS—VI.

[It is the object of these articles to show how departure from rational prescribing has fostered and encouraged a system which is open to many grievous abuses, of which self-medication is not the least. The writer appreciates the fact that many valuable preparations are sold under proprietary and protected names, and wishes it clearly to be understood that products which are recognised by medical practitioners on account of their real utility are in no way called in question.]

The quack, like the poor, we have always with us, and in spite of all efforts in the direction of reform we can never hope entirely to eliminate the charlatan from society. We can, however, by education and exposure, narrow down the *clientèle* on whom the quack levies toll, and we can do still more by legislation.

### Quackery Two Hundred Years Ago.

Two hundred years ago Sir Richard Steele wrote: "It gives me much despair in the design of reforming the world by my speculations, when I find there always arise from one generation to another successive cheats and bubbles, as naturally as beasts of prey and those who are to be their food. There is hardly a man in the world, one would think, so ignorant as not to know that the ordinary quack doctors, who publish their abilities in little brown billets, distributed to all who pass by, are to a man imposters and murderers; yet such is the credulity of the vulgar, and the impudence of these professors, that the affair still goes on, and new promises of what were never done before are made every day. What aggravates the jest, is that even this promise has been made as long as the memory of man can trace it, and yet nothing performed, and yet still prevails. As I was passing along to-day, a paper given into my hand by a fellow without a nose, tells us as follows what good news is come to town, to wit, and there is now a certain cure for the French disease, by a gentleman just come from his travels: 'In Russell Court, over against the Cannon Ball, at the Surgeon's Arms, in Drury Lane, is lately come from his travels a surgeon, who hath practised surgery and physics, both by sea and land, these twenty years. He by the blessing, cures of yellow jaundice, green sickness, scurvy, dropsy, surfeits, long sea-voyages, campaigns, &c, as some people that has been lame these thirty years can testify; in short, he cureth all diseases incident to men, women, or children.'"

During the two hundred years that have elapsed since Steele ridiculed this particular impostor, the quack has continued to flourish, and year by year, encouraged by the credulity of the ignorant, his impudent assurance has become more and more daring. The quack of two centuries ago claimed to cure "all diseases incident to men, women, and children," but there are quacks to-day who claim to cure all these ills with one and the same nostrum; there are even some whose claims are not restricted to the diseases of men, women, and children, but who hold out their "cures" as being equally beneficial to both "men and beast."

### The Responsibility of the Profession.

Even without the aid of the law, it is within the power of the medical practitioner to do much in the direction of discouraging quackery; for so long as quackery is condoned by the medical profession, it cannot be expected that laymen will condemn it. That many prescribers do—unwittingly, it is true—encourage secrecy in medicine and self medication is evident from the fact that considerably over 20 per cent. of the prescriptions written by London practitioners consist wholly or in part of branded products, a large proportion of which are compounds whose composition is known only to the makers. In the provinces, more especially in the smaller towns, the percentage of nostrums in prescriptions is considerably lower than in London; this is in a great measure due to the circumstance that the headquarters of the principal exploiters of secret remedies are situated in London, and a constant stream of plausible representatives can, at a comparatively small cost, be turned on to London practitioners to prove that what is in reality, perhaps, boric acid mixed with some substance more or less inert, and christened with a fancy name, is a newly discovered compound, the like of which has never hitherto been known.

### Quackery Above the Law.

It can be stated, without fear of contradiction, and conclusively proved by analysis, that at least 95 per cent. of all proprietary articles owe their value, if any, to some well known and readily procurable substance. Why this substance should not be ordered under its pharmacopœial name, and so made subject to the test of purity given in the British Pharmacopœia, it is difficult to understand. For when a drug or chemical is prescribed under a proprietary name, the only standard to which it need conform is that fixed by the manufacturer, who, by claiming proprietary rights, places himself outside Section VI. of the Sale of Food and Drugs Act, 1875.

Why should the physician act in the capacity of commercial traveller to the proprietary medicine manufacturer? Already we see the results, and are witnessing the humiliating spectacle of the proprietors of a series of branded products, which were introduced to the public solely through the medium of the medical prescription, now advertising them directly to the public. Apparently the physician has served the purpose of this particular firm, and the public may now doctor themselves with its products. The American Medical Association has likened the prescriber of nostrums to an "unpaid pedlar" of secret remedies, and how true this description sometimes is, may be observed every day in the numberless advertisements in the daily papers of quack medicines, of which the proprietors boast that they are "prescribed by the faculty." And sometimes this is the only statement in the whole advertisement in which there is a solitary particle of truth; dispute the statement, and the advertiser will produce properly authenticated medical prescriptions to prove his assertion.

